# Conservation genomics assessment of Tharp's bluestar (*Amsonia tharpii*) with comparisons to widespread (*A. longilora*) and narrowly endemic (*A. fugatei*) congeners

**DOI:** 10.1111/eva.13736

**Published:** 2024-06-19

**Authors:** Dylan H. Cohen, Jeremie B. Fant, Krissa A. Skogen

**Affiliations:** ^1^ Negaunee Institute for Plant Conservation Science and Action Chicago Botanic Garden Glencoe Illinois USA; ^2^ Plant Biology and Conservation Northwestern University Evanston Illinois USA; ^3^ Department of Biological Sciences Clemson University Clemson South Carolina USA

**Keywords:** *Amsonia*, conservation, genomics, land‐use change, Permian Basin, population genetics

## Abstract

Land‐use change and habitat fragmentation are threats to biodiversity. The decrease in available habitat, increase in isolation, and mating within populations can lead to elevated inbreeding, lower genetic diversity, and poor fitness. Here we investigate the genetics of two rare and threatened plant species, *Amsonia tharpii* and *A. fugatei*, and we compare them to a widespread congener *A. longiflora*. We also report the first phylogenetic study of the genus *Amsonia* (Apocynaceae), including 10 of the 17 taxa and multiple sampling locations, to understand species relationships. We used a double digest restriction‐site associated DNA sequencing (ddRADseq) approach to investigate the genetic diversity and gene flow of each species and to create a maximum likelihood phylogeny. The ddRADseq data was mapped to a reference genome to separate out the chloroplast and nuclear markers for population genetic analysis. Our results show that genetic diversity and inbreeding were low across all three species. The chloroplast and nuclear dataset in *A. tharpii* were highly structured, whereas they showed no structure for *A. fugatei*, while *A. longiflora* lacked structure for nuclear data but not chloroplast. Phylogenetic results revealed that *A. tharpii* is distinct and sister to *A. fugatei*, and together they are distantly related to *A. longiflora*. Our results demonstrated that evolutionary history and contemporary ecological processes largely influences genetic diversity within *Amsonia*. Interestingly, we show that in *A. tharpii* there was significant structure despite being pollinated by large, bodied hawkmoths that are known to be able to carry pollen long distances, suggesting that other factors are contributing to the structure observed among *A. tharpii* populations. Conservation efforts should focus on protecting all of the *A. tharpii* populations, as they contain unique genetic diversity, and a protection plan for *A. fugatei* needs to be established due to its limited distribution.

## 1 INTRODUCTION

Global change is a major cause of biodiversity decline and one of the many challenges to conservation (Haddad et al., 2015; Pereira et al., 2012; Sala et al., 2000). Pressures from land‐use change and associated habitat loss, in particular, are often compounded and accompanied by fragmentation resulting in increased isolation and changes in the configurations of habitats and populations. Fragmentation can contribute to the disruption of gene flow resulting in increased mating within populations (Haddad et al., 2015; Leimu‐Brown et al., 2010; Young et al., 1996), limiting the exchange of novel diversity between populations, and a decrease in population size. In addition, effects from climate change such as increasing temperatures and drought will lead to higher mortality rates impacting population size, especially for plants distributed in arid regions (Hantson et al., 2021; McAuliffe & Hamerlynck, 2010; Parmesan & Yohe, 2003). As a result, genetic variation may change or decrease due to small population size, inbreeding, and genetic drift. This decrease can especially be an issue for endemic species which are often rare and threatened with extinction (Corlett & Westcott, 2013; Pecl et al., 2017).

Conservation genetics approaches assess the genetic status of populations by estimating genetic diversity, population connectivity, and effective population size, which offers insight into processes impacting population dynamics for these species and guides their management (Frankham et al., 2002; Lande, 1988; Soulé, 1985; Theissinger et al., 2023). Species and populations that are small and impacted by land‐use change and fragmentation can have lower genetic diversity and increased rates of inbreeding (Aguilar et al., 2008, 2019; Cole, 2003; González et al., 2020; Honnay & Jacquemyn, 2007; Vellend, 2004), which can increase the extinction risk for threatened species through inbreeding depression, and loss of genetic diversity through drift (Brook et al., 2002; Frankham, 2005). However, gene flow (pollen or seed movement) can offset these impacts by increasing genetic variation and limiting inbreeding. Panmictic, genetically cohesive populations do not typically suffer from inbreeding depression and low genetic diversity (Charlesworth, 2003).

Populations that are connected through gene flow are potentially buffered from fragmentation (Ellstrand, 1992; Finger et al., 2014; Skogen et al., 2016). In plants, gene flow occurs through pollen and seed dispersal and the vectors of each may have distinct and different impacts on patterns of genetic diversity. Pollen transfer and seed dispersal can potentially rescue fragmented populations from low genetic diversity and inbreeding if at least one mode promotes gene flow with stable and intact populations (Frankham, 2015; Richards, 2000). Biotic pollination is known from nearly 90% of flowering plants (Ollerton et al., 2011) and the size and foraging behavior of pollinators can impact mating events and resulting patterns of genetic diversity, with larger animals carrying pollen longer distances (Gamba & Muchhala, 2020). For example, larger pollinators such as hawkmoths (Sphingidae) are capable of foraging longer distances which can create genetic cohesion among plant populations (Finger et al., 2014; Lewis et al., 2023; Skogen et al., 2019). By contrast, smaller pollinators such as bees, forage close to nesting sites and therefore over smaller areas, which can limit within and between population gene flow in the plants they visit (Dellinger et al., 2022; Greenleaf et al., 2007; Hasegawa et al., 2015). Bees also groom pollen from their bodies, limiting pollen carryover. Much like pollen movement, seed dispersal can promote connectivity (animal dispersal) or divergence (e.g. via gravity dispersal; Howe & Smallwood, 1982; Levin et al., 2003). When the mode of dispersal differs between pollen and seed dispersal, the patterns of genetic diversity will likely differ between nuclear and plastid DNA, which can help identify the main driver of gene flow in the system.

Understanding how ecological, historical, and geographic variables contribute to genetic diversity of populations and species is important for those needing management. However, it is important to recognize that the genetic patterns of a species can often be driven by evolutionary constraints and historical factors, rather than contemporary process. For these reasons, comparisons of related species are encouraged to control for evolutionary history and identify species‐specific differences (Gitzendanner & Soltis, 2000; Keller et al., 2013; Lanes et al., 2018; Ouborg et al., 2006; Soltis & Gitzendanner, 1999; Spence et al., 2021). Such approaches provide a phylogenetic perspective, which can also be critical for understanding species boundaries and can be used as a conservation framework. Closely related and recently diverged species share a common ancestor by descent and can therefore be genetically and morphologically similar. A phylogenetic approach can help to understand species relationships and species limits when genetic changes outpace morphological variation (Cohen & Schenk, 2022; Frankham, 2010; Hey et al., 2003; Purvis et al., 2005). In addition, a phylogenetic perspective can help to uncover hidden admixed or hybrid populations (Hibbins & Hahn, 2022). Furthermore, comparisons of congeners to threatened species help to determine the extent of extinction risk (Spielman et al., 2004). A phylogenetic perspective allows us to address species delimitation questions while also comparing meaningful population genetic parameters and both can be used in conservation management.

**FIGURE 1:**
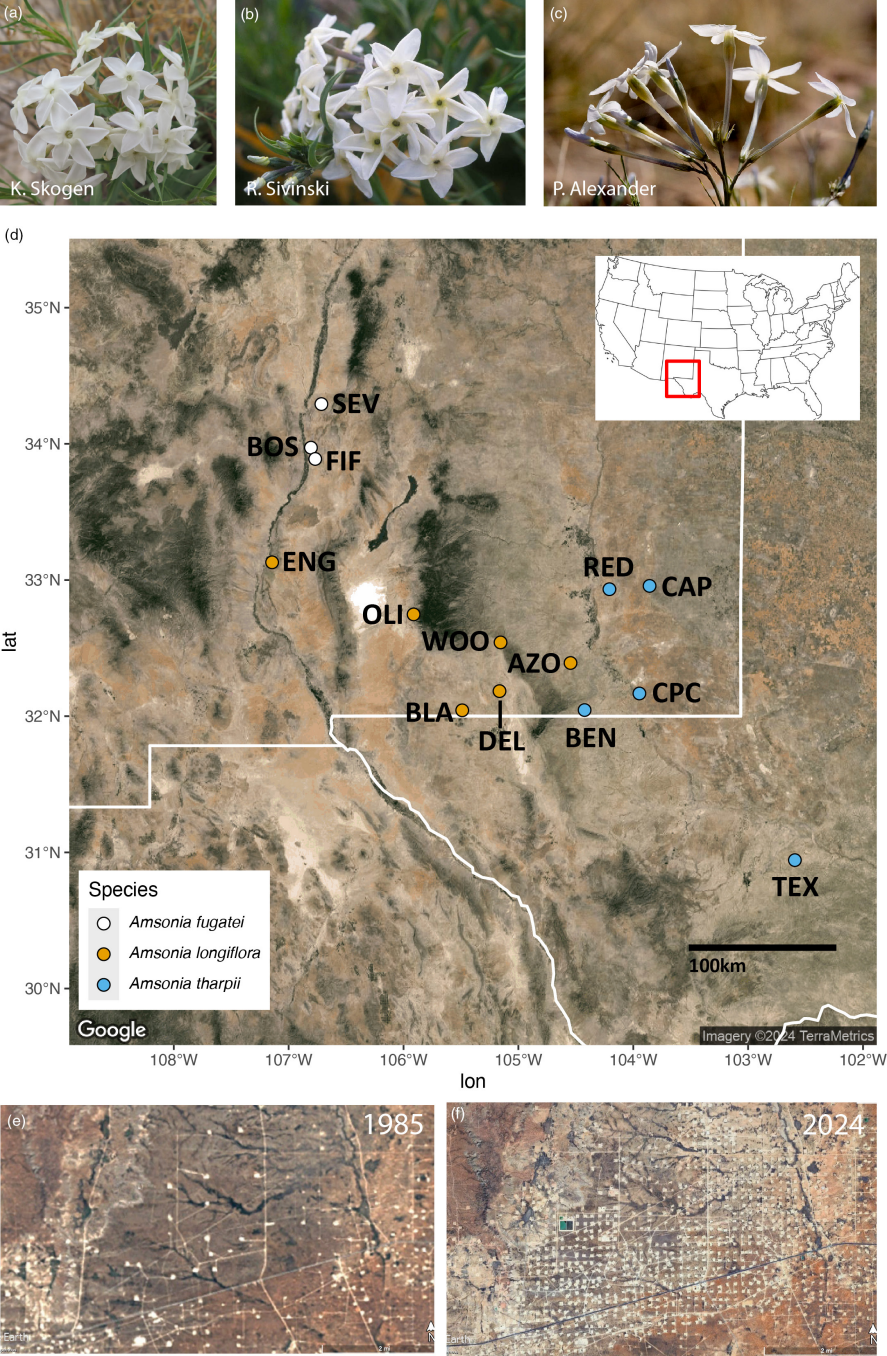
Inflorescences of (a) *Amsonia tharpii*, (b) *A. fugatei*, (c) *A. longiflora*. (d) Map of sampling localities for population genetics study. We sampled five localities from *A. tharpii*, six from *A. longiflora* and all three *A. fugatei* localities. Aerial photos of land‐use change between (e) 1985 and (f) 2024 near the Red Lake locality.

Here we compare patterns of genetic diversity, gene flow, and effective population size among three species in the plant genus *Amsonia* (Apocynaceae) to determine if rare taxa are genetically depauperate and experience limited gene flow. Two species, *A. fugatei* (Fugate's bluestar) and *A. tharpii* (Tharp's bluestar), are rare (known from three and seven localities, respectively) while *A. longiflora* (tubular bluestar) is more widespread than the other two species (Figure 1d). All three species have white, tubular flowers, with *A. longiflora* having longer floral tubes and larger corolla diameters than *A. tharpii* and *A. fugatei*, which have similar floral tube lengths and corolla diameters (Figure 1a–c). These three species occur in or near the northern extent of the Chihuahuan Desert, where much of this landscape has been impacted by land‐use change. The most dramatic change in the last 10–15 years has been a result of the extensive oil and gas development that has occurred in the Permian Basin (Figure 1e,f; Pierre et al., 2020), where all seven of the extant localities of *A. tharpii* occur (demographic monitoring by the Bureau of Land Management of the five localities included in this study started in 2017; Yannayon et al., 2021). As a result of increased pressures from oil and gas development, drought, and demographic decline, *A. tharpii* is a candidate for listing under the US Endangered Species Act. The ranges of *A. fugatei* and *A. longiflora* largely do not occur in the Permian Basin. *Amsonia fugatei* is only know from three localities and *A. longiflora* is considered secure. In addition to land‐use change, climate projections suggest that this region will experience hotter and drier conditions with increased periods of intense drought (Archer & Predick, 2008; Petrie et al., 2014). Concerns about genetic diversity in *A. tharpii* stem from demographic declines since 2017, an increase in land‐use change between localities, and climate change (Yannayon et al., 2021). In addition, because *A. tharpii* and *A. fugatei* are morphologically similar (floral tubes of similar length, similar corolla diameter; Figure 1a,b) despite being geographically isolated by the Sacramento and San Andres Mountains, we used a phylogenetic framework to elucidate species boundaries between these and other members of *Amsonia*. We hypothesize that due to the anthropogenic changes resulting in increased fragmentation in the intervening land between *A. tharpii* localities, they will have lower genetic diversity and higher inbreeding than the two secure *Amsonia* species. However, as all three species have floral traits consistent with hawkmoth pollination (white, tubular corollas), we predict that localities for each species will be genetically cohesive due to high gene flow. Indeed, other studies have shown that hawkmoths can travel long distances (Stockhouse, 1973) and population genetic studies of hawkmoth pollinated species show high rates of gene flow and little‐to‐no population differentiation (Lewis et al., 2023; Skogen et al., 2019). In addition, we hypothesize that *A. tharpii* is genetically distinct from *A. fugatei* due to geographic isolation and that *A. longiflora* is more distantly related (McLaughlin, 1982).

## 2 METHODS

### 2.1 Study system

We compared population genetics statistics from three species within *Amsonia* subg. *Sphinctosiphon* (Apocynaceae) (*A. tharpii*, *A. fugatei*, and *A. longiflora*) that vary in floral morphology, phenology, and geographic area (McLaughlin, 1982; Ngô & Applequist, 2023). *Amsonia* species are long‐lived, perennial sub‐shrubs with linear to ovate leaves, tubular white to blue corollas, and dry dehiscent fruits. Plants emerge from underground stems in early spring and typically flower in spring, and fruit in early summer. The three species have floral traits that are consistent with hawkmoth pollination: white flowers, long corolla tubes, scent (Fenster et al., 2004) and hawkmoths have been observed visiting both *A. tharpii* and *A. longiflora* (data not reported). Although the self‐compatibility system is not known for the genus, self‐incompatible systems are known from other taxa across the Apocynaceae (Coombs et al., 2009; Ollerton et al., 2019). These *Amsonia* species occur in and around the northern extent of the Chihuahuan Desert in New Mexico and west Texas on well‐drained carbonate soils such as sand, limestone, and dolomite. *Amsonia tharpii* occurs across a highly fragmented landscape in southeastern New Mexico and west Texas (Figure 1a,d–f). *Amsonia fugatei* is a narrow endemic species distributed across relatively undisturbed habitat in central Socorro County, New Mexico (Figure 1b,d, McLaughlin, 1985) and is geographically separated from *A. tharpii* by the Sacramento and San Andres Mountains. *Amsonia longiflora* has the broadest distribution extending from central New Mexico to central Texas (Figure 1c,d). Additionally, two of the *A. longifora* localities (ENG and OLI) occur west of the Sacramento Mountains and the remaining localities used in this study to the east and south of these mountains. Localities of *A. tharpi* are comprised of smaller groupings of individuals that vary in size and isolation. Samples were collected from across the known geographic extent for each of the five targeted localities.

### 2.2 Sample collection

Leaf tissue was collected in the field and dried on silica gel from of all three species during 2020 and from two *A. longiflora* localities in 2022 (BLA and OLI). We sampled 10–30 individuals per location from five localities of *A. tharpii*, all three localities of *A. fugatei*, and six localities of *A. longiflora* (Figure 1d, Table 1). To control for geographic distance, we sampled *A. longiflora* from the western edge of its range, across a geographic extent similar to the distribution of the sampled *A. tharpii* localities. We sampled from locations that occurred near one another (10–45 km) and others more distant (45–200 km) across all species except *A. fugatei* due it being limited to three localities of which the farthest were separated by 45 km (Figure 1d). Our phylogenetic assessment included eight western and two eastern North American *Amsonia* species sampled from naturally occurring localities, herbarium vouchers, and living collections at botanic gardens (Table S1).

**TABLE 1:** Locality information, individuals sampled (*N*_S_), estimated census size (*N*_C_), effective population size (*N*_e_), and inbreeding coefficient (*F*_IS_) for each locality.

Species	Locality names	Codes	*N*_S_	*N*_C_	*N*_e_	*F*_IS_
*Amsonia tharpii*	Ben Slaughter	BEN	31	122	6.5	0.010
	Cap Rock	CAP	32	1800–2800	44.9	0.070
	Cedar Pearce	CPC	33	2000–3000	63.4	0.060
	Red Lake	RED	32	4634–6734	72.8	0.088
	Texas	TEX	22	565	37.3	0.051
*A. fugatei*	Bosque	BOS	10	1000	NA	0.070
	Fife	FIF	10	500	84.8	0.096
	Sevilleta	SEV	10	600	217.2	0.060
*A. longiflora*	Azotea Mesa	AZO	10	720	NA	0.084
	Black Mountain	BLA	10	NA	43.5	0.088
	Dell City	DEL	10	50	36.3	0.043
	Engle	ENG	10	300	118.3	0.064
	Oliver	OLI	10	NA	47.5	0.083
	Woods Tank	WOO	10	NA	29.5	0.048

*Note*: The census estimates for *Amsonia tharpii* were obtained from a 2019 monitoring report (Roth & Sivinski, 2019).

### 2.3 DNA extractions/library prep

DNA extractions and two ddRADseq libraries were prepared at the Harris Genetics Laboratory at Chicago Botanic Garden. A modified CTAB protocol was used for DNA extraction (Doyle & Doyle, 1987) and all samples were visualized on electrophoresis gels and quantified with a Qubit v. 2.0 prior to library prep. Sequencing library preparation followed Peterson et al. (2012) with modifications from Diaz‐Martin et al. (2023) and others noted below. We normalized each DNA sample to 20 ng/μL prior to digestion. Normalized genomic DNA was then digested for 24 h with two restriction enzymes, EcoRI and MspI. Following digestion, P1 adapters were ligated to DNA fragments associated with the EcoRI enzyme. Libraries were multiplexed with 48 individuals (or less). After multiplexing, each library was size selected using magnetic beads or with a Pippin Prep, targeting between 300 and 650 base pairs in length. Libraries were then amplified in 20 μL PCR reactions using a thermocycler and 15–20 cycles. Libraries were cleaned with magnetic beads and quantified before sequencing at the NUSeq Core facility at Northwestern University using the Illumina NovaSeq 6000 sequencer and with 150 bp (basepairs) paired end reads.

### 2.4 Population genetics

All samples were demultiplexed, processed for mismatched sequences, cleaned, and trimmed to 100 bp with the process_radtags function in STACKS v. 2.55 (Rochette et al., 2019). Raw reads were trimmed to account for low quality and erroneous base pairs found at the end of the raw sequences. Parameters for calling SNPs were optimized using the “r80 rule” and by testing different values of *n* and *M*, while keeping *m* = 3 constant (Table S2, Paris et al., 2017). For optimization of each dataset, we first treated all the data as a single population to test different *n* and *M* values. The best parameter set was then chosen according to the most polymorphic loci and SNPs (Paris et al., 2017). We hypothesized that each location would be connected via gene flow and did not alter the –*p* value (number of populations) because this could have biased our results and called more unique loci/SNPs per population. To compare the threatened and non‐threatened species while accounting for geographic distance and evolutionary history, two different datasets were used. The first dataset, called “Individual Species SNPs”, we obtained SNPs for each taxon separately. This dataset controlled for evolutionary differences among the three species to look for population‐level divergences. The “Individual Species SNPs” dataset was separated by genome type to explore pollen and seed (nuclear genome) to only seed movement (plastid genome). To recover the plastid data from nuclear datasets we mapped demultiplexed reads to the *Amsonia tabernaemontana* reference chloroplast genome (Wang et al., 2023) using default parameters in BWA v. 0.7.12 and in SAMTOOLS v. 1.6 (Li et al., 2009; Li & Durbin, 2009). Those reads that did not map to the reference plastid genome were used for the nuclear dataset. The unmapped reads were treated as from the nuclear genome and processed in STACKS using the denovo_map.pl pipeline, whereas the mapped reads were assumed to be from the chloroplast and SNPs were called using the ref_map.pl pipeline applying the “r80 rule” and default settings.

The second dataset, “Shared SNPs”, was used to test how genetic diversity (and inbreeding) relates to evolutionary history. Little is known about the evolutionary history of *Amsonia*, but closely related and recently diverged species share many morphological and genetic attributes (McLaughlin, 1982; Ngô & Applequist, 2023). Here all three species were filtered in STACKS together, and therefore only SNPs shared across all three species were used to investigate genetic diversity and inbreeding. For all datasets, the best set of parameters was selected according to the highest number of polymorphic loci and total number of SNPs (Table S2).

VCFtools (Danecek et al., 2011) was used to filter each dataset for sequencing depth, sequence quality, minor allele frequency, and missingness before population genetic analyses. All nuclear datasets were filtered with a 90% missingness threshold allowing for only 10% missing data, a minor allele count of three, a min‐mean DP of five and a max‐mean DP of 50, a minDP of five and maxDP of 50, and maximum number of alleles of two. The plastid “Individual Species SNPs”, datasets were only filtered with a 90% missingness threshold.

Genetic diversity (expected heterozygosity) and the inbreeding coefficient (*F*_IS_) were calculated in RSTUDIO v. 4.1.2 using hierfstat v. 0.5‐11 and dartR v. 2.0.4 (Goudet, 2005; Gruber et al., 2017; R Core Team, 2021). NeEstimator v. 2 was used to calculate effective population size (*N*_e_) (Do et al., 2013). Default settings were used for NeEstimator along with a Linkage Disequilibrium model that assumed random mating events and a critical value of 0.05. Patterns of gene flow were explored from the nuclear derived datasets with pairwise *F*_ST_, isolation by distance (IBD), and with the maximum likelihood‐based program ADMIXTURE (Alexander et al., 2009). Pairwise *F*_ST_ was estimated using the Weir and Cockerham (1984) method for each locality pair across each species also using hierfstat (Goudet, 2005). We tested for correlations between population pairwise *F*_ST_ and geographic distance (IBD) using dartR and Mantel test for significance. ADMIXTURE v 1.30 was used to infer population structure (pollen movement) across each species and was run with 20 replicates testing *K* values (genetic clusters) of 1–10 for *A. tharpii* and *A. longiflora*, and 1–6 for *A. fugatei*. A 15‐fold cross‐validation (CV) was performed for each replicate, and the most appropriate *K* value was selected according to the lowest averaged CV value. ADMIXTURE results were visualized using the R package StructRly v 0.1.0 (Criscuolo & Angelini, 2020). Lastly, we used Poppr v. 2.9.4 and calculated genetic dissimilarity matrices using default settings and the bitwise.dist() function and then converted and plotted these as minimum spanning networks with poppr.msn() and plot_poppr.msn() functions (Kamvar et al., 2014, 2015) to explore plastid diversity (seed movement) within each species.

### 2.5 Phylogeny

We addressed evolutionary relationships by estimating phylogenetic relationships using 62 *Amsonia* samples from 10 of the 17 currently recognized species, and from multiple locations whenever possible, to test for species monophyly (Table S1). The Python program ipyrad v 0.9.87 (Eaton & Overcast, 2020) was used to demultiplex, filter, and cluster reads, and to align ddRAD loci for phylogenetic analysis. We clustered reads with an 85% similarity threshold and explored the effects of missing data by varying the number of samples required to have shared loci for it to be kept in the final alignment (Table S3). IQ‐Tree was used to infer a model of DNA sequence evolution and a maximum likelihood (ML) phylogeny based on the concatenated DNA sequence matrix (Nguyen et al., 2015). Model finder was used within IQ‐Tree to infer the most likely model of evolution based on AICc (Kalyaanamoorthy et al., 2017). The best fit model was then used to infer a ML tree, and node support was assessed with 100 nonparametric bootstraps (Nguyen et al., 2015). Two eastern *Amsonia* species were used as outgroups (Table S1).

## 3 RESULTS

### 3.1 Genetic diversity, inbreeding, and effective population size

**FIGURE 2:**
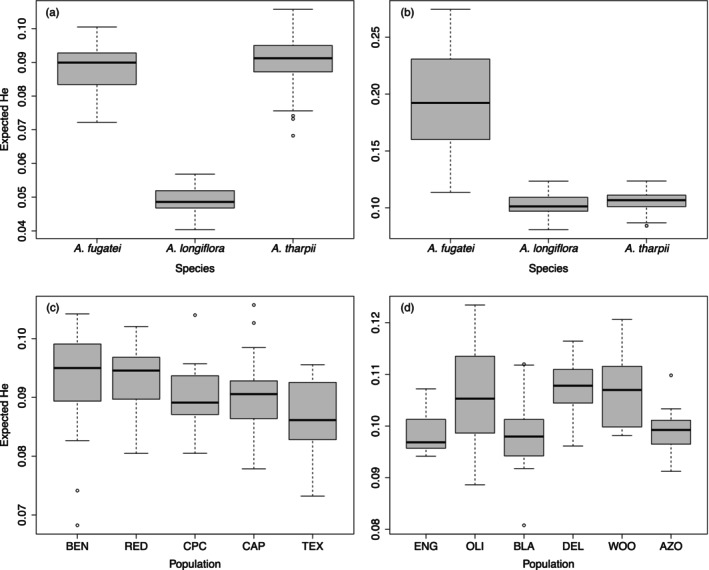
Genetic diversity (expected heterozygosity) for (a) “Shared SNPs” and (b) “Individual Species SNPs” datasets and across (c) *Amsonia tharpii* and (d) *A. longiflora* populations. “Individual Species SNPs” not shown for *A. fugatei*. Populations are plotted as they occur from west to east.

Optimization of STACKS parameters revealed that *m* = 3, *M* = 2, and *n* = 2 retrieved the most polymorphic loci and variant sites (SNPS) for the *A. tharpii* “Individual Species SNPs” dataset while *m* = 3, *M* = 2, and *n* = 3 had the most polymorphic loci and SNPs for the *A. fugatei* and *A. longiflora* datasets, and *m* = 3, *M* = 2, and *n* = 3 for the “Shared SNPs” dataset (Table S2). After filtering with VCFtools the “Individual Species SNPs” datasets recovered 1605 nuclear and 58 plastid SNPs for *A. tharpii*, 643 nuclear and 32 plastid SNPs for *A. fugatei*, and 956 nuclear and 57 plastid SNPs for *A. longiflora*. The “Shared SNPs” dataset retained 850 nuclear SNPs. Genetic diversity (expected heterozygosity) was low across both the “Individual Species SNPs” and “Shared SNPs” datasets (Figure 2). *Amsonia fugatei* and *A. tharpii* had a slightly higher genetic diversity than *A. longifora* in the “Shared SNPs” dataset and only *A. fugatei* had a higher genetic diversity than the other species in the “Individual Species SNPs” comparison (Figure 2a,b). Estimates of inbreeding were low across all species and populations (here after populations refer to genetic populations), ranging from 0.05 (*A. fugatei*) to 0.11 (*A. tharpii*) and 0.12 (*A. longiflora*) for the “Shared SNPs” datasets. Inbreeding coefficients were lower within the “Individual Species SNPs” data sets (0.06, *A. tharpii* – 0.09, *A. longiflora*), and across *A. tharpii* populations (0.01 for BEN – 0.09 for RED) and *A. longiflora* populations (0.04 for DEL – 0.09 for BLA, Table 1). Overall, effective population size was low across most populations investigated (Table 1). *Amsonia tharpii* populations had the lowest *N*_e_ and ranged from 6.5 (BEN) to 72.8 (RED). There was greater variation across *A. longiflora* populations with 29.5 (WOO) and 118.3 (ENG). *Amsonia fugatei* populations also had much higher *N*_e_ values than any of the other *Amsonia* species, ranging from 84.8 (FIF) to 217.2 (SEV, Table 1).

### 3.2 Population structure

There was no evidence of isolation by distance in any species (*A. tharpii*: *r* = 0.31, *p* = 0.34; *A. fugatei r* = 0.71, *p* = 0.33, *A. longiflora r* = 0.71, *p* = 0.07; Figure S1), however of all three species examined, *A. longiflora* had overall the lowest pairwise distance for any given distance (Table S4). Within *A. longiflora* populations the pairwise distance ranged from 0.04 (DEL and AZO, 61 km) to 0.13 (WOO and ENG, 197 km), and over a similar geographic extent, the pairwise distance of *A. tharpii* ranged from 0.08 (RED and CAP, 32 km) to 0.18 (BEN and TEX, 254 km). Last, in *A. fugatei* populations the pairwise distance (10–45 km) showed similar genetic distance, ranging from 0.08 to 0.10 (Table S4).

**FIGURE 3:**
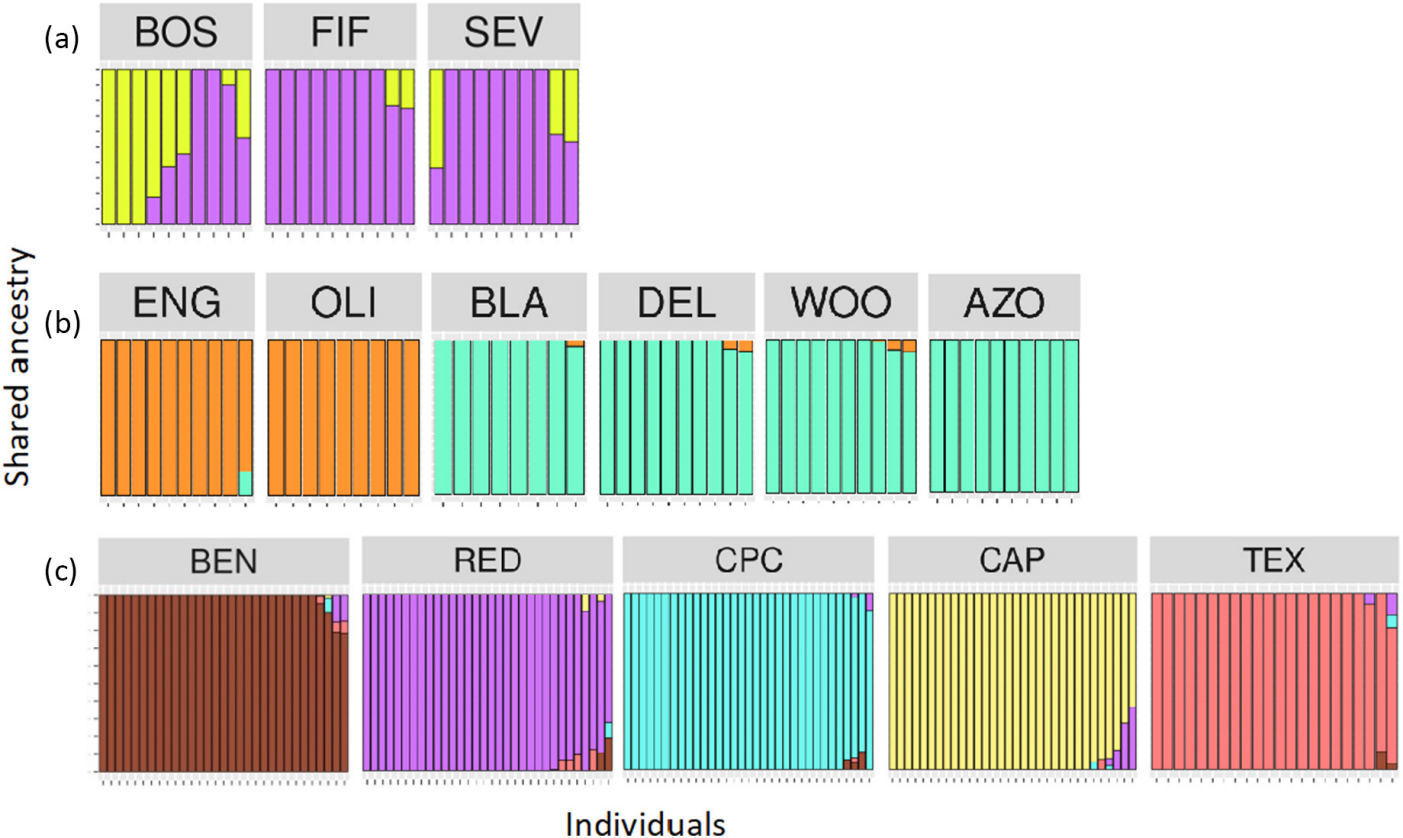
ADMIXTURE barplots for (a) *Amsonia fugatei* (*K* = 2), (b) *A. longiflora* (*K* = 2), (c) *A*. *tharpii* (*K* = 5). Populations of *A. tharpii* and *A. longiflora* are plotted from west to east as they are distributed.

**FIGURE 4:**
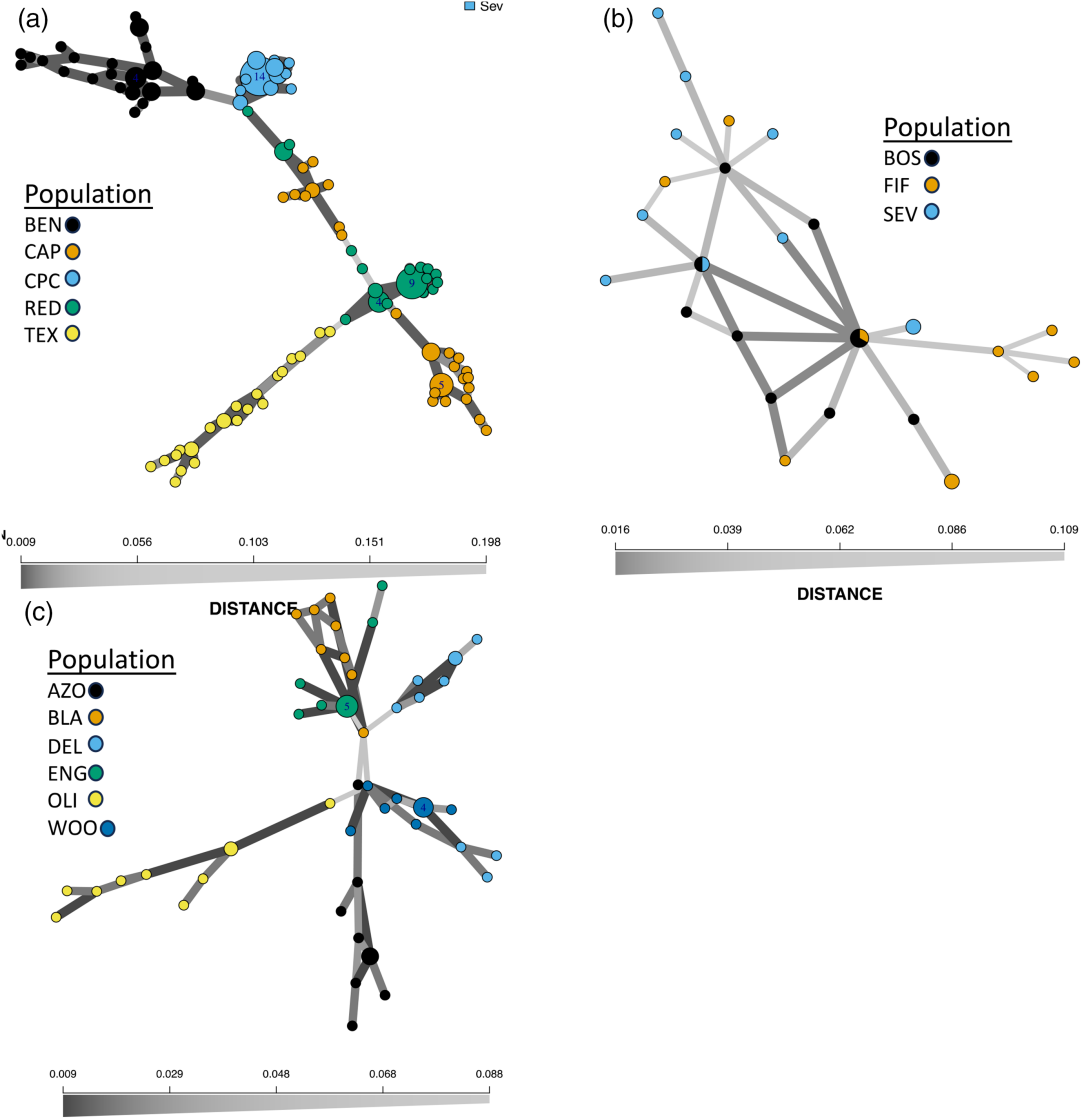
Minimum spanning networks showing plastid diversity for (a) *Amsonia tharpii*, (b) *A. fugatei*, and (c) *A. longiflora*. Numbers within circles indicate how many samples possess identical chloroplast type. Line color and width represent genetic distance between individuals and populations. Scale bar represents genetic distance.

The ADMIXTURE results suggested that *K* = 5 was the most likely number of genetic clusters for *A. tharpii* (Figure 3, Figure S2). In comparison, *A. longiflora* and *A. fugatei* cross‐validation results suggested *K* = 2 as the most likely number of genetic clusters (Figure 3, Figure S2). Within *A. longiflora* there was genetic structuring between western (ENG and OLI) and the eastern New Mexico populations (AZO, BLA, DEL, and WOO). The minimum spanning networks used to assess seed dispersal suggested that populations of *A. tharpii* and *A. longiflora* were geographically structured, however the *A. fugatei* network lacked structure (Figure 4). In addition, a few individuals from the nearest *A. tharpii* populations at RED and CAP contained similar plastid diversity that may represent an ancestral haplotype, and the TEX population appeared to be derived from the RED population (Figure 4a).

### 3.3 Phylogeny

**FIGURE 5:**
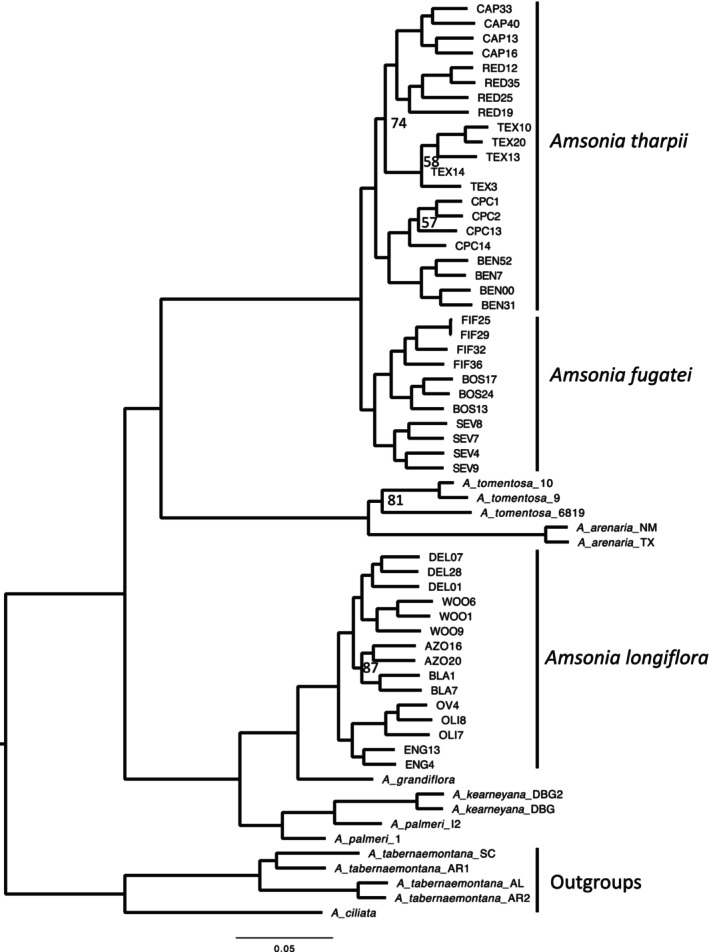
Maximum likelihood tree for *Amsonia* species. All relationships are supported (95 and above) unless indicated at nodes on phylogeny. Scale bar represents the number of nuclear substitutions along branches. *Amsonia tabernaemontana* and *A. cilata* were used to root the tree.

All three alignments retained similar topologies, however, the alignment with the most loci (50819) and missing data (61.5%) was also the best supported with bootstrap values (Table S3). The *Amsonia* backbone topology and most species relationships were strongly supported, with the only exception being *A. palmeri* (Figure 5). Two samples of *A. palmeri*, one from NM and the other from TX, were recovered as serially sister to the federally listed and narrow endemic *A. kearneyana* from southern Arizona (Figure 5). *Amsonia tharpii* was recovered as monophyletic and sister to *A. fugatei* (Figure 5). Together these species were sister to a clade that included *A. tomentosa* from the Mojave Desert and *A. arenaria* from the Chihuahuan Desert. *Amsonia longiflora* was recovered as sister to the morphologically similar *A. grandiflora* and together they were sister to *A. palmeri* and *A. kearneyana*, but distantly related to *A. tharpii* and *A. fugatei* (Figure 5). The populations of *A. tharpii*, *A. fugatei*, and *A. longiflora* were all recovered as monophyletic but some with lower support values (Figure 5).

## 4 DISCUSSION

Using a comparative approach, we showed that genetic parameters within populations were equivalent across the three species of *Amsonia* with similar floral morphology and seed traits. Genetic diversity was not lower, and inbreeding was not higher for the threatened species, *A. tharpii* or *A. fugatei*, when compared to the secure congener, *A. longiflora* (Figure 2, Table 1), however the effective population sizes for all the populations were below recommended conservation levels for all species (Table 1, Frankham et al., 2014). These results allow us to reject our hypotheses that genetic diversity is lower in the threatened *A. tharpii*. By contrast we found that there were differences in between population parameters, with *A. fugatei* and *A. longiflora* appearing to be genetically cohesive for nuclear data, likely driven by pollen movement, while *A. tharpii* showed strong structure with both nuclear and plastid genomes (Figures 3 and 4). Finally, our phylogenetic dataset supported our final hypothesis by revealing *A. tharpii* and *A. fugatei* to be genetically distinct as was the case for other members of the genus from western North America (Figure 5).

The similar levels of genetic diversity recovered across all populations and species, regardless of rarity, supports previous studies (Gitzendanner & Soltis, 2000) which suggest evolutionary history plays a large role in controlling genetic variation across closely related species (Figure 5). In comparison, a population genetic study on the eastern North American *A. ludovicana*, recovered higher genetic variation (*H*_O_ = 0.36–0.54) and similar inbreeding coefficients (*F*_IS_ = 0.01–0.13) (Figure 2, Table 1, Smallwood et al., 2018). The differences in genetic diversity could be explained by data collection method (ddRADseq vs. microsatellites) (Hodel et al., 2017; Sunde et al., 2020), sampling collection bias (Hale et al., 2012; Rosenberger et al., 2021), population sizes, or a result of being more distantly related to the western *Amsonia* species. By contrast, the inbreeding coefficients across populations of all four taxa (*A. ludovicana* and *A. tharpii*, *A. fugatei*, and *A. longiflora*) were low and consistent with a self‐incompatible breeding system, which is known in the Apocynaceae (Gibbs, 2014; Lipow & Wyatt, 1999; Shuttleworth & Johnson, 2006). Despite relatively low effective population size, similar estimates of inbreeding and genetic diversity across taxa suggest these represent historically stable population sizes. While our results suggest *A. tharpii* is not suffering from genetic erosion, individuals can be long‐lived, making it difficult to determine if the patterns revealed in our dataset represent a more contemporary or a historic legacy of gene flow. Comparisons of genetic diversity and gene flow between parent and offspring generations would better determine the impact of more recent land‐use change and resulting habitat fragmentation and extreme drought events.

The one major difference we did identify is in connectivity between populations by species, with the lowest genetic distances between populations of *A. longiflora* with the nuclear data, and for between populations of *A. fugatei* for plastid data (Table S4, Figure 4b). Because all three taxa share floral traits and hawkmoths have been recorded visiting *A. tharpii* and *A. longiflora*, (data not shown), we hypothesized that populations would be genetically similar and lack population structure across the nuclear dataset and genetically distinct (structured) in the plastid dataset. Instead, we found that *A. tharpii* was structured for both datasets (Figures 3c and 4a, Table S4). Despite the relative proximity of some *A. tharpii* populations (CAP and RED (32 km), BEN and CPC (47 km), Figure 1d, Table S4), pollen and seed movement appear to be limited to within populations. This contrasts with what other studies of hawkmoth‐pollinated taxa have found (Cisternas‐Fuentes et al., 2022; Finger et al., 2014; Rhodes et al., 2017; Skogen et al., 2019). For example, a study from the same region (Lewis et al., 2023) documented gene flow among populations of the hawkmoth pollinated *Oenothera hartwegii* subsp. *filifolia* separated by 13–400 km. This pattern is partially explained by the presence of intervening populations between the sampled populations facilitating gene flow via a steppingstone pattern. Only two known, unsampled populations occur in proximity to BEN and CPC, but even with potential steppingstone populations *A. tharpii* appears to be highly structured. Our results are consistent with other studies of plants that occupy small patches and have edaphic or unique ecological habitats whereby low genetic diversity and population structure have been documented (Barbará et al., 2008; Loveless & Hamrick, 1984; Matesanz et al., 2018; Moore et al., 2014; Ramirez‐Barahona et al., 2014) even when visited by large nectar‐feeding birds pollinators assumed to travel long distances (Bezemer et al., 2016; Nistelberger et al., 2015). While our results indicate that *A. tharpii* is experiencing genetic drift and divergence may be due to habitat fragmentation, comparisons of historic and contemporary gene flow (parent‐offspring comparisons) are needed to assess the extent to which this is likely. While the landscape surrounding sampled populations has been altered by oil and gas development in recent years, it is difficult to distinguish between its affects and those of major drought events potentially due to climate change in the region that have occurred over the same timeframe (Archer & Predick, 2008; Briggs et al., 2020; Petrie et al., 2014). The lack of adequate precipitation from annual summer monsoon events may lead to changes in population size due to mortality and delayed emergence, reductions in number of flowers, and changes in co‐flowering species diversity, which provide supplemental resources for pollinators (both floral resources for adults and vegetative for larval stages of some taxa). Over time these factors may impact population genetic parameters. Last, we cannot rule out demographic patterns, life history, and density dependent foraging patterns as a factor contributing to population structure.

One interesting outcome was that the plastid haplotype networks revealed limited seed dispersal for *A. tharpii* and *A. longifora*, but not for *A. fugatei*. Shorter seed dispersal distances are expected to increase population structure, and all *Amsonia* species have large, seeds that are primarily gravity dispersed (Gelmi‐Candusso et al., 2017; Loveless & Hamrick, 1984; Schaal et al., 1998), although their “corky” coating might suggest they have the capacity to float during flooding events. Notably, within *A. tharpii*, individuals from CAP and RED shared similar plastid diversity, and TEX appeared to be derived from the RED population, suggesting long‐distance seed movement or the existence of intervening populations at some point in the past (Figure 4a). In contrast, all three populations of *A. fugatei* shared haplotypes. *Amsonia fugatei* occurs along the Rio Grande River in central New Mexico and the lack of structure may represent a single derived haplotype or recent migration (>15,000 years ago) following extreme flooding events (Repasch et al., 2017) as seeds may be dispersed via water as seeds float.

Considering population genetic parameters in light of evolutionary history revealed similar genetic diversity and inbreeding between closely related sister species, *A. tharpii* and *A. fugatei*, and the distantly related *A. longiflora* (Figures 2 and 5). This is not surprising as comparisons of population genetic parameters between widespread and narrow endemic congeners are often correlated because of a shared derived ancestry (Gitzendanner & Soltis, 2000) and while contemporary processes impact genetic diversity measures, our results suggest this has yet to have occurred for the *A. tharpii* populations. We hypothesized that *A. tharpii* would be genetically distinct from *A. fugatei* based on geographic isolation, and our results support this (Figure 5). *Amsonia tharpii* and *A. fugatei* are separated by 250 km of distance in addition to both the Sacramento and San Andres Mountains which appeared to be the only barrier to pollen movement within *A. longiflora* populations (Figure 4). *Amsonia palmeri* was the only species recovered as non‐monophyletic (Figure 5). *Amsonia palmeri* has a wide distribution from northwestern AZ to southwestern NM, thru the Mexican states of Chihuahua and Sonora, and into eastern TX (McLaughlin, 1982; Ngô & Applequist, 2023) and additional population sampling is needed to clarify its evolutionary history. Increased sampling from the remaining, unsampled western and eastern *Amsonia* species will help to elucidate species relationships and address biogeographic patterns across the genus.

Here, we demonstrated the utility of combining a multi‐species population genetic approach with phylogenetics to inform conservation management of *A. tharpii*. Our phylogenetic results support a monophyletic *A. tharpii* that is sister to *A. fugatei*, and together they are distantly related to *A. longiflora*. These results strongly support *A. tharpii* and *A. fugatei* as distinct species and without recent gene flow or hybridization despite having similar floral and vegetative morphologies. Upon controlling for evolutionary history and geographic distance our population genetics results revealed similar genetic diversity between the two narrow endemics, *A. tharpii* and *A. fugatei*, and the widespread congener *A*. *longiflora*. In comparison, differences were found when investigating patterns of gene flow with the nuclear and plastid genomes. Even with pollination facilitated by hawkmoths, *A. tharpii* populations were structured in comparison to the lack of structure observed in *A*. *longiflora* and *A. fugatei*. Conversely the plastid analyses suggested that *A. tharpii* and *A. longiflora* to be highly structured, but the *A. fugatei* was not.

Our results can be used to inform conservation in a few specific ways. Conservation efforts should continue to focus on all populations of *A. tharpii* given they were found to be unique as they are structured. In addition, as BEN had the smallest effective and census population sizes, special attention may be warranted to ensure that this population does not go extinct. Despite less than 300 individuals occurring within a 1 km radius, genetic diversity and inbreeding estimates for BEN were similar to those of the other *A. tharpii* populations suggesting that even smaller population sizes maybe relatively stable. Furthermore, the two unsampled *A. tharpii* populations occur on private land though likely experience similar threats to the sampled populations given their close proximity to BEN and CPC, respectively. In addition, BEN and the next closest population (~12 km due east) are the only *A. tharpii* populations endemic to gypsum substrates, making them even more valuable conservation assets as they possess unique ecologies (Funk et al., 2012; Moritz, 1994). Furthermore, *A. fugatei* is known from just three populations and is restricted to a smaller geographic region than the other two species, making this species another high priority for conservation. We recommend that the Bureau of Land Management continue to monitor *A. tharpii* to track changes in population size and extent. Seed banking would provide future opportunities to restore population sizes through reintroduction if population sizes continue to decline. Additionally, oil and gas development that has occurred in close proximity to *A. tharpii* populations should not encroach further.

## CONFLICT OF INTEREST STATEMENT

There are no conflicts of interests to declare.

## Supporting information


Figure S1.

Figure S2.

Table S1.

Table S2.

Table S3.

Table S4.

## Data Availability

Raw sequencing data for this study are available at: https://www.ncbi.nlm.nih.gov/bioproject/1106052. Further scripts to recreate analyses and additional output files used for those analyses are located here: https://github.com/dylanHco/Amsonia‐Popgen/tree/main.
