# Supplementary figures and images for: Conservation genomics assessment of Tharp's bluestar (Amsonia tharpii) with comparisons to widespread (A. longilora) and narrowly endemic (A. fugatei) congeners
Source: Evol Appl. 2024 Jun 19;17(6):e13736. doi: 10.1111/eva.13736 (PMC11186748; doi:10.1111/eva.13736)

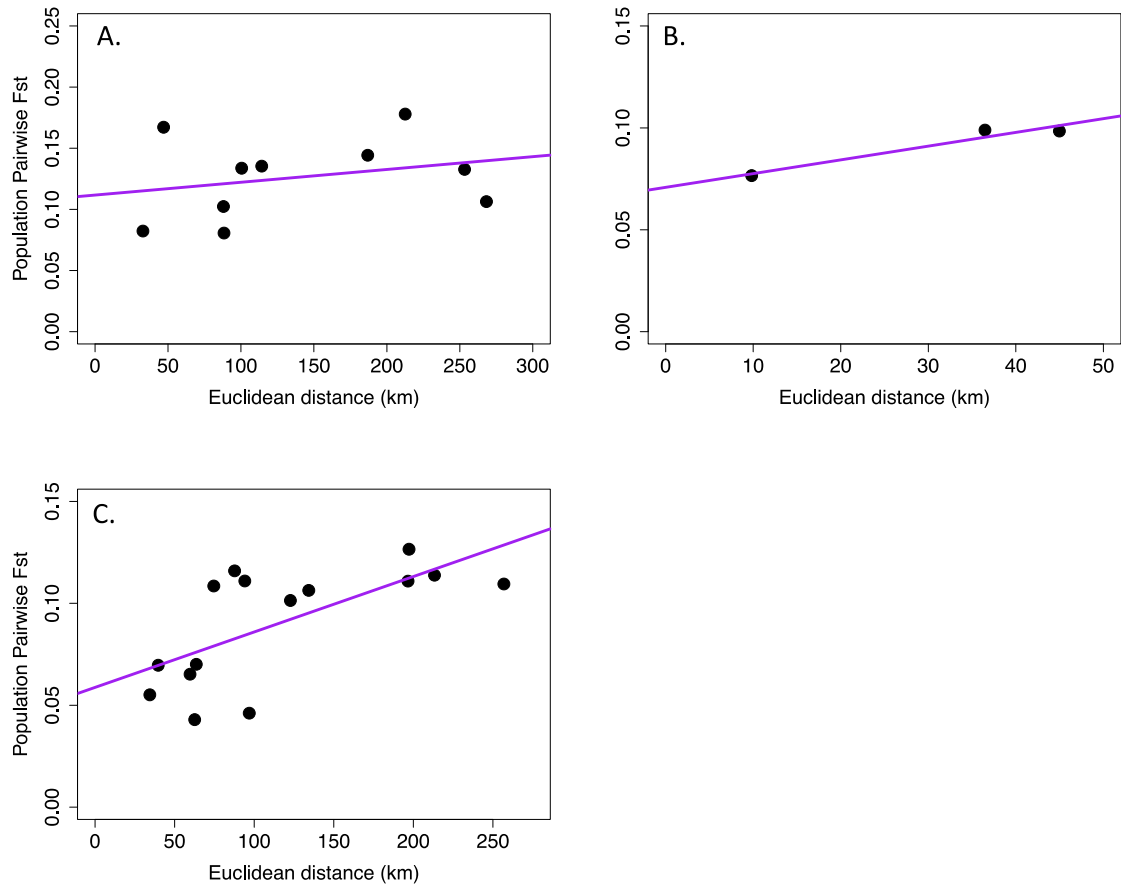

Figure S1. Isolation by distance scatter plot for A) *Amsonia tharpii*, B) *A. fugatei*, and C) *A. longiflora*.

Supplement: Supplementary file 1 — Figure S1. [file EVA-17-e13736-s003.pdf]
