# Supplementary material for: Conservation genomics assessment of Tharp's bluestar (Amsonia tharpii) with comparisons to widespread (A. longilora) and narrowly endemic (A. fugatei) congeners
Source: Evol Appl. 2024 Jun 19;17(6):e13736. doi: 10.1111/eva.13736 (PMC11186748; doi:10.1111/eva.13736)
Supplement: Supplementary file 2 — Figure S2. [file EVA-17-e13736-s001.pdf]

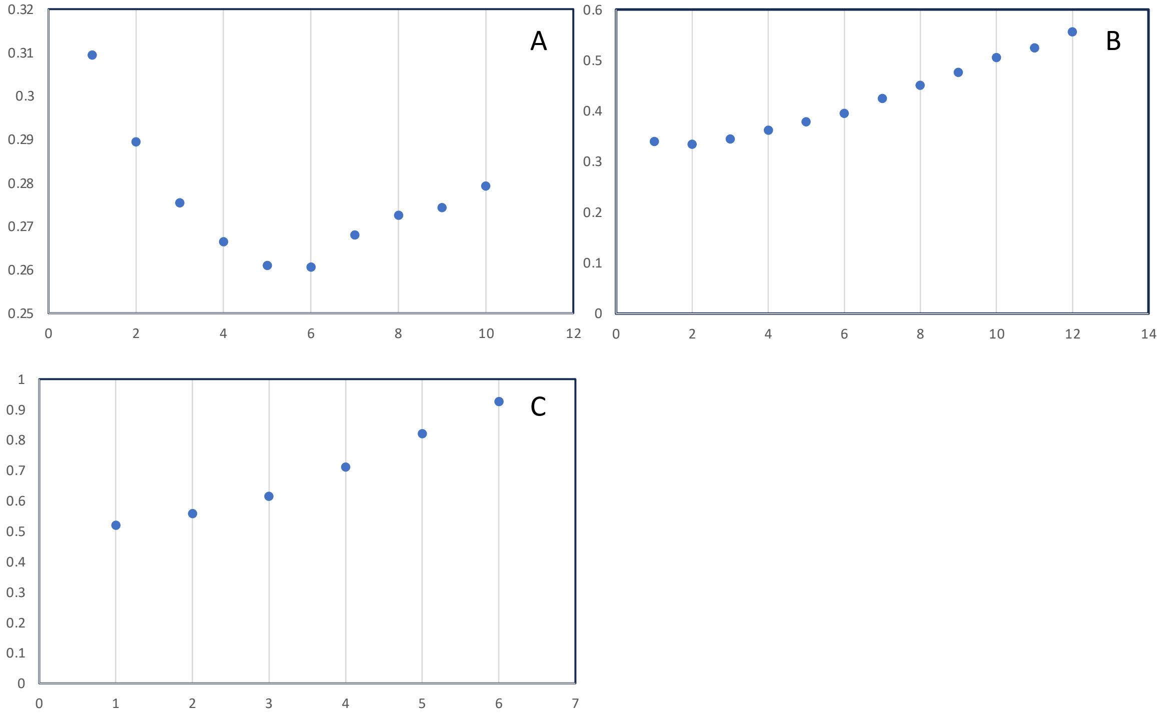

Figure S2. ADMIXTURE cross validation results for a) *Amosnia tharpii*, b) *A. longiflora*, and c) *A. fugatei*. The best K value was chosen according to the lowest cross validation score.
