# Supplementary material for: Conservation genomics assessment of Tharp's bluestar (Amsonia tharpii) with comparisons to widespread (A. longilora) and narrowly endemic (A. fugatei) congeners
Source: Evol Appl. 2024 Jun 19;17(6):e13736. doi: 10.1111/eva.13736 (PMC11186748; doi:10.1111/eva.13736)
Supplement: Supplementary file 3 — Table S1. [file EVA-17-e13736-s004.docx]

Table S1. Species and number of samples included for phylogenetic tree reconstruction. Outgroups are bolded. Samples collected from herbaria are indicated in parentheses.

| Species | # of samples | Subgenus | State collected | Obtained from |
| --- | --- | --- | --- | --- |
| *Amsonia arenaria* | 2 | *Articularia* | NM/TX | Field |
| ***A. ciliata*** | 1 | *Amsonia* | SC | Field |
| *A. fugatei* | 11 | *Sphinctosiphon* | NM | Field |
| *A. grandiflora* | 1 | *Sphinctosiphon* | AZ | Desert Botanic Garden |
| *A. kearneyana* | 2 | *Sphinctosiphon* | AZ | Desert Botanic Garden |
| *A. longiflora* | 15 | *Sphinctosiphon* | NM | Field |
| *A. palmeri* | 2 | *Sphinctosiphon* | AZ/NM | Field/Herbarium (F) |
| ***A. tabernaemontana*** | 4 | *Amsonia* | AL/AR/SC | Field/Herbarium (CLEM) |
| *A. tharpii* | 21 | *Sphinctosiphon* | NM/TX | Field |
| *A. tomentosa* | 3 | *Articularia* | CA/NV | Field/Herbarium (F) |
