# Supplementary material for: Conservation genomics assessment of Tharp's bluestar (Amsonia tharpii) with comparisons to widespread (A. longilora) and narrowly endemic (A. fugatei) congeners
Source: Evol Appl. 2024 Jun 19;17(6):e13736. doi: 10.1111/eva.13736 (PMC11186748; doi:10.1111/eva.13736)
Supplement: Supplementary file 4 — Table S2. [file EVA-17-e13736-s002.docx]

Table S2. Optimization of STACKS parameters for population genetic analyses. We report the total number of variant sites (SNPs) and polymorphic loci for each parameter set. Best parameters are in bold.

| Stacks parameters  m-M-n | *A. tharpii* | *A. fugatei* | *A. longiflora* | Shared SNPs |
| --- | --- | --- | --- | --- |
| 3-2-1 | 25391/28066 | 15238/16251 | 18355/18675 | 10876/11198 |
| 3-1-1 | 26887/29910 | 15451/16548 | 18826/19161 | 11073/11012 |
| 3-2-2 | **26954/29731** | 15635/16679 | 19286/19622 | 12318/12440 |
| 3-3-3 | 24300/26859 | 15246/16278 | 18652/19022 | 12586/12713 |
| 3-4-4 | 23353/25836 | 14863/15913 | 18083/18457 | 12572/12702 |
| 3-3-2 | 24343/26949 | 15147/16149 | 18508/18858 | 12108/12242 |
| 3-2-3 | 25404/28043 | **15837/16931** | **19361/19756** | **12956/13094** |
