# Supplementary material for: Conservation genomics assessment of Tharp's bluestar (Amsonia tharpii) with comparisons to widespread (A. longilora) and narrowly endemic (A. fugatei) congeners
Source: Evol Appl. 2024 Jun 19;17(6):e13736. doi: 10.1111/eva.13736 (PMC11186748; doi:10.1111/eva.13736)
Supplement: Supplementary file 5 — Table S3. [file EVA-17-e13736-s005.docx]

Table S3. Optimization of ipyrad parameters for phylogenetic reconstruction. The number of shared loci required to be present in samples was adjusted by adjusting the min_samples_locus parameter.

| Alignment Name | Min loci | Total Loci | Percent missing data |
| --- | --- | --- | --- |
| Amo_4 | 4 | 50819 | 61.5 |
| Amo_30 | 30 | 10682 | 25.3 |
| Amo_45 | 45 | 6155 | 13.8 |
