# Supplementary material for: Conservation genomics assessment of Tharp's bluestar (Amsonia tharpii) with comparisons to widespread (A. longilora) and narrowly endemic (A. fugatei) congeners
Source: Evol Appl. 2024 Jun 19;17(6):e13736. doi: 10.1111/eva.13736 (PMC11186748; doi:10.1111/eva.13736)
Supplement: Supplementary file 6 — Table S4. [file EVA-17-e13736-s006.docx]

Table S4 Pairwise Fst comparison (bottom) and geographic distance in km (top) for A) *A. tharpii*, B) *A. fugatei*, and C) *A. longiflora*.

B.

A.

| BEN |  | 47 | 112 | 88 | 209 |
| --- | --- | --- | --- | --- | --- |
| CPC | 0.144 |  | 84 | 76 | 185 |
| CAP | 0.167 | 0.123 |  | 32 | 248 |
| RED | 0.134 | 0.094 | 0.081 |  | 254 |
| TEX | 0.178 | 0.144 | 0.144 | 0.106 |  |

| BOS |  | 10 | 36 |
| --- | --- | --- | --- |
| FIF | 0.077 |  | 45 |
| SEV | 0.099 | 0.098 |  |

C.

| AZO |  | 95 | 61 | 255 | 132 | 57 |
| --- | --- | --- | --- | --- | --- | --- |
| BLA | 0.046 |  | 33 | 195 | 89 | 66 |
| DEL | 0.043 | 0.055 |  | 212 | 94 | 44 |
| ENG | 0.109 | 0.111 | 0.114 |  | 122 | 197 |
| OVI | 0.106 | 0.116 | 0.111 | 0.101 |  | 72 |
